# A Gram-Negative Bacterial Secreted Protein Types Prediction Method Based on PSI-BLAST Profile

**DOI:** 10.1155/2016/3206741

**Published:** 2016-08-02

**Authors:** Shuyan Ding, Shengli Zhang

**Affiliations:** ^1^Department of Sciences, Dalian Nationalities University, Dalian 116600, China; ^2^School of Mathematics and Statistics, Xidian University, Xi'an 710071, China

## Abstract

Prediction of secreted protein types based solely on sequence data remains to be a challenging problem. In this study, we extract the long-range correlation information and linear correlation information from position-specific score matrix (PSSM). A total of 6800 features are extracted at 17 different gaps; then, 309 features are selected by a filter feature selection method based on the training set. To verify the performance of our method, jackknife and independent dataset tests are performed on the test set and the reported overall accuracies are 93.60% and 100%, respectively. Comparison of our results with the existing method shows that our method provides the favorable performance for secreted protein type prediction.

## 1. Introduction

Protein secretion is a universal and important biological process and it can occur in both eukaryotes and prokaryotes. In recent years, several secreted proteins have been identified as markers for disease typing and staging [1, 2] or the development of drugs [3]. Most bacteria are able to secrete proteins, such as toxins and hydrolytic enzymes, into the extracellular environment. In this process, Gram-negative bacterial proteins have to be transported across the two lipid bilayers, including the cytoplasmic membrane (CM) and the outer membrane (OM) [4]. Proteins, including virulence factors involved in invasion, colonization, and survival within a host organism, are produced in pathogenic Gram-negative bacteria and are secreted to the cell exterior [5]. They play different roles in invaded eukaryotic cells and cause various diseases [4], so it is important to study them for the pathogenesis of diseases and the development of drugs.

Secretion systems are capable of specifically recognizing their substrates and facilitating secretion without disturbing the barrier function of the cell envelope. However, they differ tremendously with respect to their functional mechanism and complexity. So far, eight secretion systems have been found in Gram-negative bacteria and named from the type I (T1SS) to the type VIII secretion system (T8SS) according to the OM secretion mechanisms [4]. Correspondingly, proteins released via the T1SS are called type I secreted proteins (T1SPs), and other types of proteins are named by analogy with this.

In fact, prediction of protein datasets such as protein structural classes prediction and Subcellular localization prediction is a typical and traditional pattern recognition problem. Generally, it can be performed in three main steps: feature extraction, feature selection, and model selection for classification. Among the three steps, feature extraction is the most critical and challenging step for the prediction. Amino acid composition (AAC) [6–9], pseudoamino acid composition (PseAAC) [10–12], polypeptide composition [13], functional domain composition [14], PSI-BLAST profile [15, 16], and so on are all the widely used feature extraction methods. In order to reduce the computation complexity and pick out the more informative features, a feature selection step is necessary. Principal component analysis (PCA) [17], SVM-RFE [18], and correlation-based feature selection (CFS) [19] have performed well in the feature selection. Finally, choosing a powerful classification tool is also very important. Neural network [8], support vector machine (SVM) [9, 20], fuzzy clustering [21], and rough sets [22] are usually being used.

In 2013, Yu et al. constructed a dataset of Gram-negative bacterial secreted proteins which contains 839 secreted proteins [23]. The proteins are collected from three data sources, namely, SwissProt, TrEMBL [24], and RefSeq [25]. They used an improved PseAAC consisting of amino acid composition (AAC) and autocovariance (AC) to extract information from PSI-BLAST profile. The support vector machine (SVM) is used to distinguish different types of secreted proteins in their paper and the reported highest overall accuracy of their method is 90.12%.

Recently, some researchers try to improve the prediction accuracy of protein datasets by combining the dipeptide composition and PSI-BLAST profile together [15, 16, 26–28]. These methods mainly focused on the single-column information extraction based on the hypothesis that two neighboring amino acids are independent which may make the neighboring correlation information lost.

In this study, we also extracted the evolutionary information from PSI-BLAST profile based on correlation method to perform Gram-negative bacterial secreted proteins prediction. A feature set consisting of 309 features is selected by correlation-based feature selection (CFS) method based on training set. With the selected 309 features, the jackknife test and independent test are performed on test set by SVM. The results show that our method is reliable for the secreted protein type prediction.

## 2. Materials and Methods

### 2.1. Materials

Yu et al. constructed a dataset of Gram-negative bacterial secreted proteins which contains 839 secreted proteins with 25% similarity. The dataset is divided into training set and test set. The 667 secreted proteins belong to training set and the other 172 secreted proteins belong to test set. The protein numbers of each type are listed in Table 1. In fact, 16 T6SPs and 24 T8SPs were also collected from several data sources as shown in the paper of Yu et al.; however, owing to the small numbers and high sequence similarity, they are just suitable for phylogenetic analysis to understand the evolutionary history [23]. Hence, only six types of Gram-negative bacterial secreted proteins are considered. The datasets can be downloaded from http://web.xidian.edu.cn/slzhang/paper.html.

### 2.2. Feature Extraction

PSI-BLAST profile is usually denoted by a position-specific score matrix (PSSM) which includes abundant evolutionary information. PSSM is calculated by applying the PSI-BLAST [29] in which three iterations are used and its cut off value is set to 10^−6^ on SwissProt dataset. Given a protein sequence, PSSM produces the substitution probability of the amino acids along its sequence based on their position with all 20 amino acids. PSSM is a log-odds matrix of size *L* × 20, where *L* is length of the query amino acid sequence and 20 is due to the 20 amino acids. The (*i*, *j*)th entry of the matrix represents the score of the amino acid in the *i*th position of the query sequence being mutated to amino acid type *j* during the evolution process.

In this study, the PSSM elements are scaled to the range from 0 to 1 using the following sigmoid function:

(1) $$\begin{array}{c} f\left(\;x\;\right)=\frac{1}{1+e^{-x}}, \end{array}$$

where *x* is the original PSSM value.

For convenience, we denote

(2) $$\begin{array}{c} D=\left(\;P_{1},P_{2},\ldots ,P_{20}\;\right) \end{array}$$

as the PSSM of the query sequence *S* with length *L*, where, for example,

(3) $$\begin{array}{c} P_{j}={\left(\;p_{1,j},p_{2,j},\ldots ,p_{L,j}\;\right)}^{T}. \end{array}$$

*T* is the transpose operator, and *p*_*i*,*j*_  (*i* = 1,2,…, *L*) denotes the score of the amino acid in the *i*th position of *S* being mutated to the *j*th amino acid during the evolution process.

In our previous study, we combine the long-range correlation information and linear correlation information of *P*_*s*_ and *P*_*t*_  (*s* ≠ *t*) together to perform the feature extraction and the linear correlation coefficient of (*p*_1,*s*_, *p*_2,*s*_,…,*p*_*L*−*g*,*s*_)^*T*^ and (*p*_*g*+1,*t*_, *p*_*g*+2,*t*_,…,*p*_*L*,*t*_)^*T*^ is used to reflect the average correlation between two residues separated by a gap of *g* along the sequence *S* [30]. For convenience, for a fixed *g*, we list the formulae as follows:

(4) As,t,g=1L−g∑i=1L−gpi,s×pi+g,t,Bs,g=1L−g∑i=1L−gpi,s,Ct,m=1L−m∑i=g+1Lpi,t,Ds,g=1L−g∑i=1L−gpi,s2−1L−g∑i=1L−gpi,s2,Et,g=1L−g∑i=g+1Lpi,t2−1L−g∑i=g+1Lpi,t2.

Then, we define

(5) $$\begin{array}{c} {\text{LCC}}_{s,t,g}=\frac{A_{s,t,g}-B_{s,g}\times C_{t,g}}{\sqrt{D_{s,g}\times E_{t,g}}}. \end{array}$$

For a fixed *g*, we define

(6) Ψg=LCC1,1,g,LCC1,2,g,…,LCC1,20,g,LCC2,1,g,…,LCC2,20,g,…,LCC20,20,g.

Ψ_*g*_ is a 400-dimensional vector, where *g* = 0,1, 2,….

Suppose that the maximum value of *g* is *G*; then the feature vector can be denoted by

(7) $$\begin{array}{c} F={\left(\;\Psi_{0},\Psi_{1},\ldots ,\Psi_{g},\ldots ,\Psi_{G}\;\right)}^{T}. \end{array}$$

The dimension of feature vector *F* is 400*∗*(*G* + 1). However, there may exist some irrelevant and redundant information among the extracted features, which can lead to a poor prediction. Hence, a feature selection method is used.

### 2.3. Feature Selection and the Selection of *G*

Feature selection can reduce the dimensionality of the data and may allow learning algorithms to operate faster and more effectively. Wrapper and filter are two main directions developed for feature selection. In order to determine the value of *G*, CFS method [19] is performed to the (*G* + 1)*∗*400 features to filter out poorly informative ones with *G* varying from 0 to 16. As shown in Hall's paper, as a filter method, in many cases CFS gave comparable results to the wrapper and, in general, outperformed the wrapper on small datasets [19].

**Figure 1:**
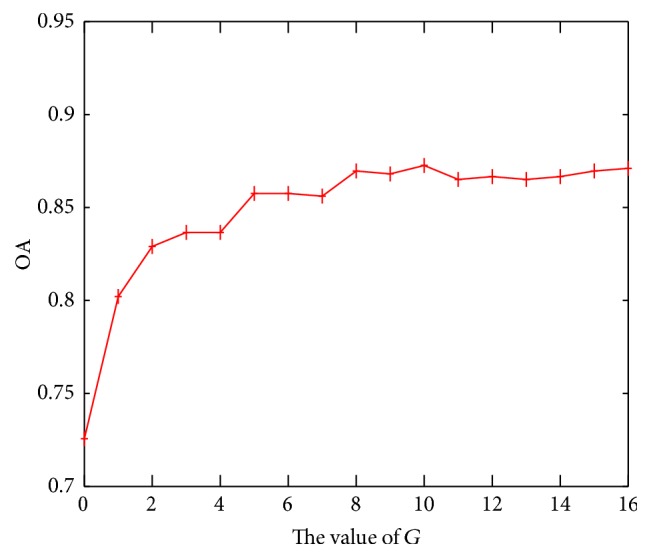
The overall accuracy of training dataset with *G* ranging from 0 to 16.

Then, the jackknife test is performed on the training set based on the selected features. The overall accuracies of training set at different values of *G* are shown in Figure 1, from which we can find that the highest overall accuracy of training set is achieved at *G* = 10. Hence, in this paper, *G* is set to be 10. The selected feature numbers with the varies of *g* when *G* = 10 are listed in Table 2. From Table 2, it is found that when *g* = 2, the selected features are the most which arrives at 45. While when *g* = 5,10, only 18 features are selected. When *g* is bigger than 10, the long-range correlation of residues becomes more and more weak with *g* increases. This is consistent with the phenomenon shown in Figure 1 that the overall accuracy becomes stable when *G* is bigger than 10.

### 2.4. Classification Algorithm Construction

SVM can often achieve superior classification performance in comparison with other classification algorithms. In this study, the support vector machine (SVM) classifier is employed as the classification algorithm. The radial basis function (RBF) is selected as the kernel function, which is defined as

(8) $$\begin{array}{c} K\left(\;x_{i},x_{j}\;\right)=e^{-\gamma {\left\|x_{i}-x_{j}\right\|}^{2}}, \end{array}$$

where *γ* is a kernel parameter and *x*_*i*_ and *x*_*j*_ are the feature vector of the *i*th and *j*th proteins, respectively. The regularization parameter *C* (used to control the trade-off between allowing training errors and forcing rigid margins) and kernel parameter *γ* are optimized based on tenfold cross-validation on training set. *C* is allowed to take a value of 2^−5^, 2^−4^,…, 2^0^, 2^1^,…, 2^15^ and *γ* to take a value of 2^−15^, 2^−14^,…, 2^0^, 2^1^,…, 2^5^. Various pairs of (*C*, *γ*) values are tried and the one with the best cross-validation accuracy is picked. The final classifier uses *C* = 4096 and *γ* = 0.5.

## 3. Prediction Assessment

Independent dataset test, subsampling test, and jackknife test are usually used to examine the effectiveness of a predictor in statistical prediction. The jackknife test and independent dataset test are used to examine the power of our method. The standard performance measures including the sensitivity (Sens), specificity (Spec), overall accuracy (OA), and Matthew's correlation coefficient (MCC) are used to evaluate the prediction accuracy. The MCC value ranges between −1 and 1, where 0 represents random correlation, and bigger positive (negative) values indicate better (lower) prediction quality for a given class [31]. Explicitly, they are defined by the following formulas:

(9) Sens=TPTP+FNSpec=TNFP+TNOA=TP+TNTP+FN+FP+TNMCC=TP×TN−FP×FNTP+FPTP+FNTN+FPTN+FN,

where TP is the number of true positives, FP is the number of false positives, TN is the number of true negatives, and FN is the number of false negatives, respectively.

## 4. Results

To evaluate the performance of our method, jackknife test was performed on training set and test set, respectively. The detailed prediction results are listed in Table 3. The overall accuracies are both higher than 85%. If comparing the six types to each other, the prediction of T1SP and T5SP types is both higher than 90% for the training set. For the training set, the prediction accuracy of T4SP is only 67.74%, which may be due to the unbalance of this dataset. For the test set, the accuracies of other four types are all higher than 90% excluding T1SP and T4SP types. Excluding T4SP type, the MCC values of the other five types are all higher than 0.9 which shows that our method is effective for the Gram-negative bacterial secreted protein types prediction.

In addition, the independent dataset test is performed on test set. The method is trained by SVM based on training set; then the obtained model is used to perform the prediction of test set. An excellent result is obtained and all the types are predicted correctly and the result is shown in Table 4. The overall accuracy of 100% is obtained by our method for the test data. Compared with the result of Yu et al. [23] obtained by “one-to-one” algorithm, the overall accuracy obtained by our method is 9.88% higher than that of Yu's method. Compared with the “one-to-the-rest” algorithm result of Yu's method (2013), the overall accuracy of our method is 13.95% higher.

The result shows that the extracted information, especially the information extracted from different columns of PSSM, plays an important role in the improvement of the prediction accuracy. In addition, the combined information extracted at different gaps *g* can provide more useful information for the prediction.

## 5. Conclusions

In recent years, more and more secreted proteins have been discovered from a variety of Gram-negative bacteria. Hence, how to determine the type of new discovered Gram-negative bacterial secreted protein is becoming an urgent research task. A set which contains six types of Gram-negative bacterial secreted proteins was constructed by Yu et al. in 2013. In this paper, the long-range correlation information and linear correlation information are extracted from position-specific score matrix (PSSM). The best optimal residue distance is determined based on the training set. Results by jackknife test and independent dataset test on the test set show that our method is effective in predicting Gram-negative bacterial secreted protein types.

## Figures and Tables

**Table 1:** The protein numbers of each type in training set and test set.

Type	Training set	Test set
T1SP	112	25
T2SP	99	29
T3SP	182	28
T4SP	62	22
T5SP	164	35
T7SP	48	33

**Table 2:** The selected feature numbers for training set at *G* = 10 (*g* ranges from 0 to 10).

The value of *g*	0	1	2	3	4	5	6	7	8	9	10
Number of selected features	35	36	45	30	22	18	33	28	22	22	18

**Table 3:** The prediction quality of our method on training set and test set.

Dataset	Class	Sens (%)	Spec (%)	MCC
Training set	T1SP	91.07	99.64	0.94
	T2SP	79.80	97.18	0.78
	T3SP	89.01	89.90	0.76
	T4SP	67.74	98.35	0.72
	T5SP	96.34	99.20	0.96
	T7SP	81.25	99.35	0.85
	OA	87.26		

Test set	T1SP	84.00	100.0	0.90
	T2SP	100.0	97.90	0.94
	T3SP	92.86	98.61	0.92
	T4SP	86.36	98.67	0.87
	T5SP	97.14	99.27	0.96
	T7SP	96.97	97.84	0.93
	OA	93.60		

**Table 4:** The comparison of our prediction quality with Yu's method by independent dataset test on the test set.

Type	Reference	T1SP	T2SP	T3SP	T4SP	T5SP	T7SP	Total
Number of sequences		25	29	28	22	35	33	172
The “one-to-one” algorithm								
Correct hit		22	23	28	18	35	29	155
Sensitivity (%)		88.00	79.31	100.00	81.82	100.00	87.88	90.12

The “one-to-the-rest” algorithm								
Correct hit	[23]	20	22	28	17	34	27	148
Sensitivity (%)	[23]	80.00	75.86	100.00	77.27	97.14	81.82	86.05

Correct hit	Our method	25	29	28	22	35	33	172
Sensitivity (%)	Our method	100.0	100.0	100.0	100.0	100.0	100.0	100.0
